# A Randomised Cross-Over Pharmacokinetic Bioavailability Study of Synthetic *versus* Kiwifruit-Derived Vitamin C

**DOI:** 10.3390/nu5114451

**Published:** 2013-11-11

**Authors:** Anitra C. Carr, Stephanie M. Bozonet, Margreet C. M. Vissers

**Affiliations:** Centre for Free Radical Research, Department of Pathology & Biomedical Science, University of Otago, Christchurch, P.O. Box 4345, Christchurch 8140, New Zealand; E-Mails: stephanie.bozonet@otago.ac.nz (S.M.B.); margreet.vissers@otago.ac.nz (M.C.M.V.)

**Keywords:** human, ascorbic acid, ascorbate, plasma, urine, kiwi fruit

## Abstract

Kiwifruit are a rich source of vitamin C and also contain numerous phytochemicals, such as flavonoids, which may influence the bioavailability of kiwifruit-derived vitamin C. The aim of this study was to compare the relative bioavailability of synthetic *versus* kiwifruit-derived vitamin C using a randomised cross-over pharmacokinetic study design. Nine non-smoking males (aged 18–35 years) received either a chewable tablet (200 mg vitamin C) or the equivalent dose from gold kiwifruit (*Actinidia chinensis* var. *Sungold*). Fasting blood and urine were collected half hourly to hourly over the eight hours following intervention. The ascorbate content of the plasma and urine was determined using HPLC with electrochemical detection. Plasma ascorbate levels increased from 0.5 h after the intervention (*P* = 0.008). No significant differences in the plasma time-concentration curves were observed between the two interventions (*P* = 0.645). An estimate of the total increase in plasma ascorbate indicated complete uptake of the ingested vitamin C tablet and kiwifruit-derived vitamin C. There was an increase in urinary ascorbate excretion, relative to urinary creatinine, from two hours post intervention (*P* < 0.001). There was also a significant difference between the two interventions, with enhanced ascorbate excretion observed in the kiwifruit group (*P* = 0.016). Urinary excretion was calculated as ~40% and ~50% of the ingested dose from the vitamin C tablet and kiwifruit arms, respectively. Overall, our pharmacokinetic study has shown comparable relative bioavailability of kiwifruit-derived vitamin C and synthetic vitamin C.

## 1. Introduction

In contrast to most mammals, humans cannot synthesis vitamin C (ascorbate) due to mutation of the terminal biosynthetic enzyme [1]. Thus, the micronutrient must be obtained from dietary sources in order to prevent hypovitaminosis C and the potentially fatal deficiency disease scurvy [1]. Vitamin C was first isolated from fruit and vegetables and the adrenal glands of animals in the early 1930s and was chemically synthesized in 1933 [2]. Although synthetic and food-derived vitamin C is chemically identical, fruit and vegetables contain numerous nutrients and phytochemicals, e.g., flavonoids, which may affect the bioavailability of food-derived vitamin C. Flavonoids can act as antioxidants via direct scavenging of free radicals [3] and/or chelation of redox-active metal ions [4,5]. Thus, it has been proposed that food-derived flavonoids may “spare” vitamin C and thus increase its bioavailability.

Due to the low bioavailability of flavonoids [6] and tight sequestration of metal ions *in vivo* [7], this vitamin C “sparing” mechanism may be expected to occur primarily in the intestinal lumen. Vitamin C is actively transported through the intestinal epithelium via the sodium-dependent vitamin C transporter 1 (SVCT1) [8]. This transporter is also responsible for renal reabsorption of vitamin C, which helps to maintain whole body homeostasis [9]. SVCT1 has a higher capacity, but lower affinity, for vitamin C than the SCVT2 isoform, which is found in most other metabolically active cells and tissues [9].

Although food matrix interactions can influence the bioavailability of some nutrients, such as carotenoids [10], the bioavailability of vitamin C does not appear to be influenced by the food matrix. Kamp *et al.* [11] found no difference in vitamin C bioavailability from a micronutrient supplement administered in the absence or presence of a corn-based porridge. Mangels *et al*. [12] also found no difference between vitamin C bioavailability from oranges compared with orange juice, and although there was a difference in bioavailability between raw and cooked broccoli, this was likely due to differences in mechanical homogenization (chewing), a similar effect to that observed for carotenoid absorption from raw *versus* cooked carrots. 

Vitamin C bioavailability can be determined using either steady-state or pharmacokinetic study designs. The former monitors ascorbate levels in blood, cells, tissues and/or urine following a number of weeks of supplementation, while the latter monitors transient changes in plasma levels and/or urinary excretion over the hours following ingestion of the test substance. We have carried out a steady state bioavailability study comparing synthetic with kiwifruit-derived vitamin C in healthy non-smoking males supplemented with a vitamin C tablet or the equivalent dose of vitamin C from gold kiwifruit [13]. No differences in steady state bioavailability were observed in plasma, urine, semen, leukocytes, or muscle tissue following six weeks of supplementation, despite significant differences being observed in our earlier animal model study [14].

Transient differences between synthetic vitamin C and that from different fruit juices have been observed using pharmacokinetic models [15,16,17,18]. Therefore, the aim of the current study was to compare the relative bioavailability of synthetic *versus* kiwifruit-derived vitamin C using a randomised cross-over pharmacokinetic study design to determine whether there are any transient differences between the two interventions. Uptake of vitamin C exhibits sigmoidal steady state kinetics between doses of 30–400 mg/day, with plasma uptake decreasing at doses of >200 mg/day and urinary excretion increasing at doses ≥100 mg/day [19]. Therefore, we chose a dose of 200 mg vitamin C and the comparable dose derived from gold kiwifruit (*Actinidia chinensis* var. *Sungold*). Our study participants were young non-smoking men with “healthy” (*i.e.*, >50 µmol/L) baseline levels of plasma vitamin C.

## 2. Study Design and Methods

### 2.1. Participants

All procedures involving human participants were approved by the New Zealand Health and Disability Ethics Committee (#URA/11/02/003). Non-smoking males aged 18–35 years were recruited from our previous vitamin C study databases, and were primarily from local tertiary institutes. Exclusion criteria included: recent smoker (within previous year), low plasma vitamin C (<50 µmol/L), allergy/intolerance to kiwifruit, taking prescription medication (within past three months), and fainting due to fear of needles. Anthropometric measurements were carried out to determine body mass index (BMI) and a fasting venous blood sample was drawn to determine plasma ascorbate levels as described below. Nine participants with above average plasma ascorbate levels were enrolled for the study and provided written informed consent. 

### 2.2. Study Design

**Figure 1:**
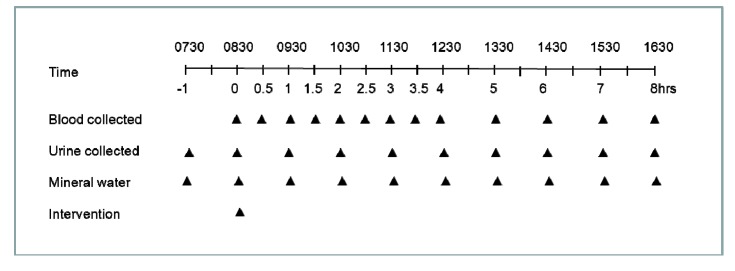
Pharmacokinetic study design. Participants received either 1.5 *Sungold* kiwifruit (containing ~200 mg vitamin C) or chewable vitamin C tablets (total of 200 mg vitamin C) in a randomised cross-over design with a three week washout period between the two clinic days.

The randomised cross-over pharmacokinetic study design comprised eight hours of sampling after a single dose of 1.5 *Sungold* kiwifruit (containing ~200 mg vitamin C) or chewable vitamin C tablets (total of 200 mg vitamin C) with a three-week washout period between the two clinic days (Figure 1) [20]. Following an overnight fast, the participants had a venous cannula inserted and blood samples were collected at baseline, and every 30 min post-intervention for the first four hours, then every hour for the next four hours. The cannulae were flushed with up to 10 mL sterile saline after each sampling. Urine was collected at baseline, after emptying the bladder an hour earlier, and then every hour post-intervention for the next eight hours. Mineral water (75 mL) was provided every hour and a cheese sandwich at four hours post-intervention. One of the nine participants did not carry out the vitamin C tablet supplementation arm of the study.

### 2.3. Interventions

Chewable orange-flavoured vitamin C tablets were provided by Tishcon Corp., Westbury, NY, USA. Analysis of the tablets indicated that they contained 49 mg of vitamin C per tablet. Participants received a single dose of four tablets, equivalent to ~196 mg vitamin C.

Gold kiwifruit (*Actinidia chinensis* var. *Sungold*) were provided by Zespri International Ltd., Mount Maunganui, New Zealand, and were stored at ≤4 °C. The vitamin C content of the kiwifruit was monitored by HPLC with electrochemical detection [21]. HPLC analysis indicated there was 162 ± 18 mg vitamin C per 100 g fruit (*n* = 5). Participants consumed 1.5 kiwifruit, without the skin (*i.e.*, ~78% of 103 ± 8.4 g fruit); therefore the actual amount of vitamin C consumed was estimated to be ~194 mg per 1.5 kiwifruit.

### 2.4. Sample Collection and Processing

*Plasma.* Peripheral blood was collected into 4 mL K_3_-EDTA vacutainer tubes, which were kept on ice at all times. Samples were centrifuged at 4 °C to pellet cells and the plasma was collected and kept on ice for extraction of ascorbate. Plasma samples were treated with an equal volume of ice-cold 0.54 M perchloric acid containing the metal chelator DTPA (100 µmol/L) to precipitate protein [21]. The de-proteinated supernatants were stored at −80 °C until HPLC analysis.

*Urine.* Urine was collected into pre-weighed 50 mL sample containers and an aliquot was removed for urinary creatinine determinations. Urine samples were treated with an equal volume of ice-cold 0.54 M perchloric acid containing the metal chelator DTPA (100 µmol/L) to precipitate protein [21]. The de-proteinated supernatants were stored at −80 °C until HPLC analysis.

### 2.5. Plasma and Urine Analysis

*Creatinine.* Urinary creatinine was determined by the Jaffe reaction using an Abbot c8000 analyser (Canterbury Health Laboratories, Christchurch, New Zealand).

*Ascorbate.* The ascorbate content of the plasma and urine was analysed using reverse-phase HPLC, with a Synergi 4 µ Hydro-RP 80A column and an ESA Coulochem II electrochemical detector, as previously described in detail [13]. In order to measure total ascorbate (reduced and oxidised), duplicate samples were reduced with tris(2-carboxyethyl)phosphine hydrochloride as described previously [22]. 

### 2.6. Data and Statistical Analysis

Area under the plasma and urinary ascorbate time-concentration curve calculations were determined using the trapezoidal rule as described previously [23]. The increase in circulating ascorbate (by weight) was calculated using Nadler’s formula for estimating total blood volume [24] and the increase in urinary ascorbate (by weight) was calculated from the total volume of urine collected. Data are represented as mean ± SD for group characteristics or mean ± SEM for comparisons between group means. Analysis of paired data was determined using two-tailed Students *t-*test with *P* <0.05 indicating statistical difference. Two way analysis of variance with Fisher pairwise multiple comparison procedure was carried out using SigmaStat software (version 11, Systat Software Inc., San Jose, CA, USA).

## 3. Results

### 3.1. Participant Characteristics

At screening, the participants’ mean ± SD age was 24 ± 5 years, weight was 78 ± 10 kg, height was 180 ± 8 cm, and BMI was 24 ± 2 kg/m^2^. The participants selected for this study had “healthy” (*i.e.*, >50 µmol/L) fasting plasma ascorbate concentrations, their mean ± SD at screening being 67 ± 17 µmol/L (*n* = 9). This was to avoid the potentially confounding effects of preferential tissue uptake in individuals with suboptimal ascorbate status at baseline, which could affect the comparative ascorbate levels observed in plasma and urine. Urinary ascorbate levels were detectible in the subjects at baseline, further indicating that plasma ascorbate levels were at or above the renal threshold.

### 3.2. Vitamin C Uptake into Plasma

**Figure 2:**
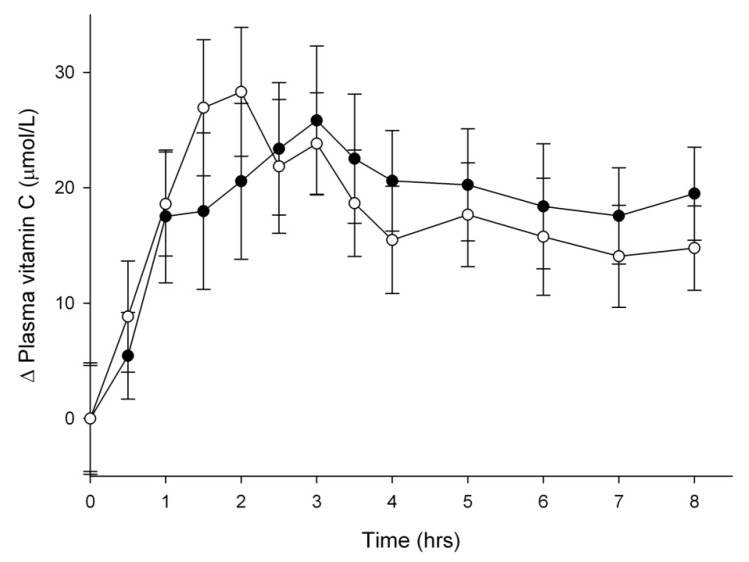
Change in plasma ascorbate uptake following ingestion of 200 mg vitamin C (●) or 1.5 Sungold kiwifruit (○). Data represent mean ± SEM (*n* = 9). Baseline plasma ascorbate concentrations were 61 ± 6 µmol/L and 66 ± 6 µmol/L for the vitamin C and kiwifruit groups, respectively. Two way analysis of variance with Fisher pairwise multiple comparison procedure indicated a significant increase in plasma ascorbate from 0.5 h post intervention (*P* = 0.008), but no significant difference between the two interventions (*P* = 0.645).

Plasma ascorbate levels, following ingestion of either 200 mg vitamin C tablets or 1.5 Sungold kiwifruit (providing an equivalent dose of ~200mg vitamin C), are shown in Figure 2. A statistically significant increase in plasma ascorbate was observed as early as 0.5 h post intervention (*P* = 0.008). No significant differences in the plasma time-concentration curves were observed between the two interventions (*P* = 0.645). Area under the ascorbate time-concentration curves also indicated no difference between the two interventions (Table 1). An estimate of the total increase in plasma ascorbate indicated that all of the ingested vitamin C tablet and kiwifruit-derived vitamin C accumulated in plasma over the eight-hour time course (Table 1).

**Table 1:** Area under the plasma and urinary ascorbate time-concentration curves (AUC) and total increase in plasma and urinary ascorbate. Subjects were supplemented with 200 mg vitamin C or 1.5 Sungold kiwifruit and ascorbate concentrations in plasma and urine were determined over the eight hours post intervention.

	Vitamin C (200 mg) ^a^	Kiwifruit (1.5 Sungold) ^a^	*P* value ^b^
Plasma AUC (h × µmol/L)	220 ± 23	237 ± 13	0.483
Plasma ascorbate (mg) ^c^	211 ± 18	227 ± 16	0.496
Urinary AUC (h × µmol/mmol creatinine)	618 ± 133	856 ± 118	0.004
Urinary ascorbate (mg)	74 ± 14	101 ± 10	0.033

^a^ Data represent mean ± SEM; ^b^ *P* values were determined using paired two-tailed Students *t*-test. ^c^ Total blood volumes were estimated using Nadler’s formula [24].

### 3.3. Vitamin C Excretion in Urine

**Figure 3:**
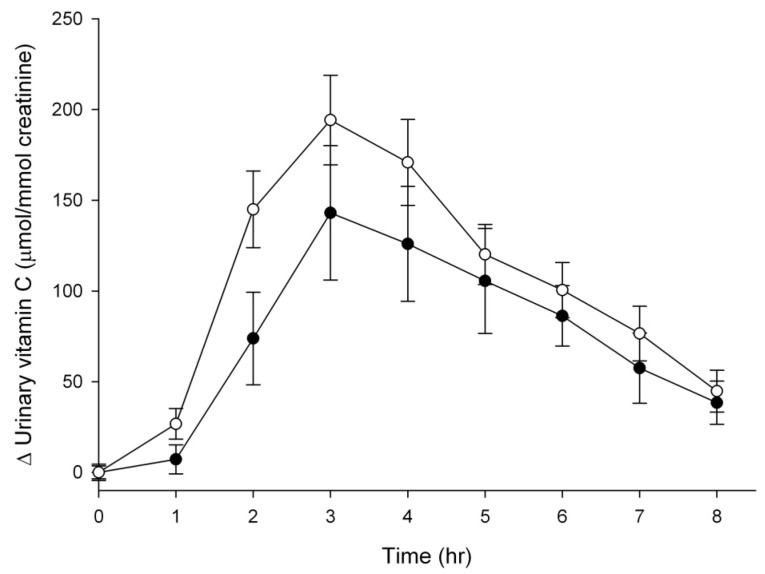
Change in urinary ascorbate excretion following ingestion of 200 mg vitamin C (●) or 1.5 Sungold kiwifruit (○). Data represent mean ± SEM (*n* = 9). Baseline urinary ascorbate concentrations were 10 ± 4 µmol/mmol creatinine and 14 ± 3 µmol/mmol creatinine for the vitamin C and kiwifruit groups, respectively. Two way analysis of variance with Fisher pairwise multiple comparison procedure indicated a significant increase in plasma vitamin C from two hours post intervention (*P* < 0.001), as well as a significant difference between the two interventions (*P* = 0.016).

Urinary ascorbate excretion was monitored over the eight hours following intervention with vitamin C tablets or Sungold kiwifruit and standardised to urinary creatinine concentrations (Figure 3). There was a significant increase in ascorbate excretion from two hours post intervention (*P* < 0.001) and also a significant difference between the two interventions, with enhanced ascorbate excretion observed in the kiwifruit group (*P* = 0.016, Figure 3). This difference was confirmed with ascorbate area under the time-concentration curves (Table 1). The total increase in ascorbate excretion over the eight hours indicated ~40% and ~50% excretion of the ingested dose from the supplement and kiwifruit arms, respectively (Table 1).

## 4. Discussion

Our pharmacokinetic study has shown comparable plasma uptake of synthetic and kiwifruit-derived vitamin C in subjects with “healthy” (*i.e.*, >50 µmol/L) baseline ascorbate status. Several other pharmacokinetic studies have shown comparable plasma uptake of vitamin C supplied in synthetic form *versus* that in fruit juices [15,23,25]. Nelson *et al.* [26] used an intestinal triple lumen tube perfusion model to investigate the absorption of synthetic vitamin C and that from an orange juice solution. This method allowed direct measurement of intraluminal events and showed no difference in the absorption of vitamin C from the two test solutions. Some pharmacokinetic studies have shown small or transient decreases in plasma ascorbate levels in the presence of fruit juices [16,18]. The physiological relevance of these small or transient differences is, however, likely minimal.

Vinson and Bose are the only investigators to have shown increased uptake of vitamin C, in the presence of citrus fruit extract, using a pharmacokinetic study design [27,28]. Their initial study, carried out in guinea pigs, indicated that vitamin C provided in a citrus fruit medium took longer to reach peak plasma concentrations compared with a synthetic vitamin C solution and also provided a larger area under the plasma ascorbate concentration-time curve [27]. These investigators also observed a comparable trend in human subjects supplemented with 500 mg vitamin C in the presence or absence of a citrus fruit extract [28]. The citrus extract delayed maximal plasma levels by one hour and provided a 35% increase in vitamin C levels.

The different outcomes observed in the pharmacokinetic studies discussed above could be explained by differences in study design and subjects. For example, the doses of vitamin C used varied by more than 10-fold, from 30 mg to 500 mg. Vitamin C bioavailability is non-linear [29] and although ~100% bioavailability is observed at a vitamin C dose of 200 mg, doses exceeding this exhibit decreased intestinal uptake [19]. Doses of 500 mg vitamin C are also significantly higher than would be obtained through a normal daily diet. Furthermore, the participants in these pharmacokinetic studies exhibited varying baseline plasma ascorbate levels, from 35 µmol/L to 75 µmol/L, the former being non-saturating and the latter being saturating levels of ascorbate [21]. These different baseline levels may affect vitamin C uptake and clearance kinetics as it is likely that there will be preferential uptake into cells and tissues in individuals with suboptimal vitamin C status [21,30]. Thus, it is possible that kiwifruit-derived vitamin C may exhibit different uptake kinetics in individuals with sub-optimal ascorbate status at baseline.

We observed a transient increase in urinary excretion when ascorbate was supplied as kiwifruit compared with tablets. Vinson and Bose [28] observed increased excretion of ascorbate when given in the presence of citrus fruit extract, but only in individuals who had been saturated with vitamin C prior to beginning the study. Similarly, others have shown small or transient increases in ascorbate excretion in the presence of fruit juice in pre-saturated subjects [17,18]. In contrast, Uchida *et al*. [15] recently reported decreased ascorbate excretion when given in the presence of acerola juice. Decreased excretion of fruit-derived vitamin C was observed in subjects with low baseline ascorbate status [15,28], supporting the premise that baseline ascorbate status may affect comparative vitamin C bioavailability. 

The mechanism whereby kiwifruit enhances urinary excretion of ascorbate without affecting plasma levels is unkown. Certain dietary fibres, such as hemicellulose, which is present in kiwifruit [31], have been shown to increase the excretion of ascorbate [32]. The flavonoid quercetin, found in kiwifruit [33], is a reversible, non-competitive inhibitor of ascorbate transport by SVCT1 [34]. Due to the low intestinal bioavailabiltiy of flavonoids [6], this mechanism would be expected to occur primarily in the intestinal lumen. Although we did not observe an effect of kiwifruit on plasma uptake of ascorbate, alternative mechanisms for intestinal uptake have been implicated using SVCT1 knockout mice [35]. An alternative, hypothetical mechanism may involve a kiwifruit-derived metabolite, which is excreted into urine and thus selectively inhibits ascorbate reabsorption via SVCT1 in the kidney tubules. Urinary excretion of ascorbate may be advantageous with respect to diseases or infections of the urinary tract [36,37].

## 5. Conclusions

Our pharmacokinetic study has shown comparable plasma uptake of synthetic and kiwifruit-derived vitamin C and enhanced urinary excretion of kiwifruit-derived vitamin C, although the ~10% increase in total excretion is unlikely to be physiologically significant. Of interest, in our subjects with >50 µmol/L to saturating baseline plasma ascorbate, ~50%–60% of the ingested ascorbate was unaccounted for by urinary excretion, despite complete plasma uptake. This indicates possible tissue uptake even in individuals with supposedly “healthy” or “optimal” plasma ascorbate status.

## References

[1] Tsao C.S., Packer L., Fuchs J. (1997). An Overview of Ascorbic Acid Chemistry and Biochemistry. Vitamin C in Health and Disease.

[2] Sauberlich H.E., Packer L., Fuchs J. (1997). A History of Scurvy and Vitamin C. Vitamin C in Health and Disease.

[3] Ivanov V., Carr A.C., Frei B. (2001). Red wine antioxidants bind to human lipoproteins and protect them from metal ion-dependent and -independent oxidation. J. Agric. Food Chem..

[4] Beker B.Y., Sonmezoglu I., Imer F., Apak R. (2011). Protection of ascorbic acid from copper(II)-catalyzed oxidative degradation in the presence of flavonoids: Quercetin, catechin and morin. Int. J. Food Sci. Nutr..

[5] Clemetson C.A., Andersen L. (1966). Plant polyphenols as antioxidants for ascorbic acid. Ann. N. Y. Acad. Sci..

[6] Lotito S.B., Frei B. (2006). Consumption of flavonoid-rich foods and increased plasma antioxidant capacity in humans: Cause, consequence, or epiphenomenon?. Free Radic. Biol. Med..

[7] Carr A., Frei B. (1999). Does vitamin C act as a pro-oxidant under physiological conditions?. Faseb J..

[8] Tsukaguchi H., Tokui T., Mackenzie B., Berger U.V., Chen X.Z., Wang Y., Brubaker R.F., Hediger M.A. (1999). A family of mammalian Na^+^-dependent l-ascorbic acid transporters. Nature.

[9] Savini I., Rossi A., Pierro C., Avigliano L., Catani M.V. (2008). SVCT1 and SVCT2: Key proteins for vitamin C uptake. Amino Acids.

[10] Parada J., Aguilera J.M. (2007). Food microstructure affects the bioavailability of several nutrients. J. Food Sci..

[11] Kamp F., Jandel D., Hoenicke I., Pietrzk K., Gross R., Trugo N.M., Donangelo C.M. (2003). Bioavailability of iron, zinc, folate, and vitamin C in the IRIS multi-micronutrient supplement: Effect of combination with a milk-based cornstarch porridge. Food Nutr. Bull..

[12] Mangels A.R., Block G., Frey C.M., Patterson B.H., Taylor P.R., Norkus E.P., Levander O.A. (1993). The bioavailability to humans of ascorbic acid from oranges, orange juice and cooked broccoli is similar to that of synthetic ascorbic acid. J. Nutr..

[13] Carr A.C., Bozonet S.M., Pullar J.M., Simcock J.W., Vissers M.C. (2013). A randomised steady-state bioavailability study of synthetic and natural (kiwifruit-derived) vitamin C. Nutrients.

[14] Vissers M.C.M., Bozonet S.M., Pearson J.F., Braithwaite L.J. (2011). Dietary ascorbate affects steady state tissue levels in vitamin C-deficient mice: Tissue deficiency after sub-optimal intake and superior bioavailability from a food source (kiwifruit). Am. J. Clin. Nutr..

[15] Uchida E., Kondo Y., Amano A., Aizawa S., Hanamura T., Aoki H., Nagamine K., Koizumi T., Maruyama N., Ishigami A. (2011). Absorption and excretion of ascorbic acid alone and in acerola (*Malpighia emarginata*) juice: Comparison in healthy Japanese subjects. Biol. Pharm. Bull..

[16] Bates C.J., Jones K.S., Bluck L.J. (2004). Stable isotope-labelled vitamin C as a probe for vitamin C absorption by human subjects. Br. J. Nutr..

[17] Jones E., Hughes R.E. (1984). The influence of bioflavonoids on the absorption of vitamin C. IRCS Med. Sci..

[18] Pelletier O., Keith M.O. (1974). Bioavailability of synthetic and natural ascorbic acid. J. Am. Diet. Assoc..

[19] Levine M., Conry-Cantilena C., Wang Y., Welch R.W., Washko P.W., Dhariwal K.R., Park J.B., Lazarev A., Graumlich J.K., King J., Cantilena L.R. (1996). Vitamin C pharmacokinetics in healthy volunteers: Evidence for a recommended dietary allowance. Proc. Natl. Acad. Sci. USA.

[20] Kondo Y., Higashi C., Iwama M., Ishihara K., Handa S., Mugita H., Maruyama N., Koga H., Ishigami A. (2012). Bioavailability of vitamin C from mashed potatoes and potato chips after oral administration in healthy Japanese men. Br. J. Nutr..

[21] Carr A.C., Pullar J.M., Moran S., Vissers M.C.M. (2012). Bioavailability of vitamin C from kiwifruit in non-smoking males: Determination of ‘healthy’ and ‘optimal’ intakes. J. Nutr. Sci..

[22] Sato Y., Uchiki T., Iwama M., Kishimoto Y., Takahashi R., Ishigami A. (2010). Determination of dehydroascorbic acid in mouse tissues and plasma by using tris(2-carboxyethyl)phosphine hydrochloride as reductant in metaphosphoric acid/ethylenediaminetetraacetic acid solution. Biol. Pharm. Bull..

[23] Carter B., Monsivais P., Drewnowski A. (2010). Absorption of folic acid and ascorbic acid from nutrient comparable beverages. J. Food Sci..

[24] Nadler S.B., Hidalgo J.H., Bloch T. (1962). Prediction of blood volume in normal human adults. Surgery.

[25] Guarnieri S., Riso P., Porrini M. (2007). Orange juice *vs.* vitamin C: Effect on hydrogen peroxide-induced DNA damage in mononuclear blood cells. Br. J. Nutr..

[26] Nelson E.W., Streiff R.R., Cerda J.J. (1975). Comparative bioavailability of folate and vitamin C from a synthetic and a natural source. Am. J. Clin. Nutr..

[27] Vinson J.A., Bose P. (1983). Comparative bioavailability of synthetic and natural vitamin C in guinea pigs. Nutr. Rep. Int..

[28] Vinson J.A., Bose P. (1988). Comparative bioavailability to humans of ascorbic acid alone or in a citrus extract. Am. J. Clin. Nutr..

[29] Graumlich J.F., Ludden T.M., Conry-Cantilena C., Cantilena L.R., Wang Y., Levine M. (1997). Pharmacokinetic model of ascorbic acid in healthy male volunteers during depletion and repletion. Pharm. Res..

[30] Carr A.C., Bozonet S.M., Pullar J.M., Simcock J.W., Vissers M.C. (2013). Human skeletal muscle ascorbate is highly responsive to changes in vitamin C intake and plasma concentrations. Am. J. Clin. Nutr..

[31] Sims I.M., Monro J.A. (2013). Fiber: Composition, structures, and functional properties. Adv. Food Nutr. Res..

[32] Keltz F.R., Kies C., Fox H.M. (1978). Urinary ascorbic acid excretion in the human as affected by dietary fiber and zinc. Am. J. Clin. Nutr..

[33] Lee D.E., Shin B.J., Hur H.J., Kim J.H., Kim J., Kang N.J., Kim D.O., Lee C.Y., Lee K.W., Lee H.J. (2010). Quercetin, the active phenolic component in kiwifruit, prevents hydrogen peroxide-induced inhibition of gap-junction intercellular communication. Br. J. Nutr..

[34] Song J., Kwon O., Chen S., Daruwala R., Eck P., Park J.B., Levine M. (2002). Flavonoid inhibition of sodium-dependent vitamin C transporter 1 (SVCT1) and glucose transporter isoform 2 (GLUT2), intestinal transporters for vitamin C and glucose. J. Biol. Chem..

[35] Corpe C.P., Tu H., Eck P., Wang J., Faulhaber-Walter R., Schnermann J., Margolis S., Padayatty S., Sun H., Wang Y., Nussbaum R.L., Espey M.G., Leine M. (2010). Vitamin C transporter Slc23a1 links renal reabsorption, vitamin C tissue accumulation, and perinatal survival in mice. J. Clin. Investig..

[36] Yalcin O., Karatas F., Erulas F.A., Ozdemir E. (2004). The levels of glutathione peroxidase, vitamin A, E, C and lipid peroxidation in patients with transitional cell carcinoma of the bladder. BJU Int..

[37] Ochoa-Brust G.J., Fernandez A.R., Villanueva-Ruiz G.J., Velasco R., Trujillo-Hernandez B., Vasquez C. (2007). Daily intake of 100 mg ascorbic acid as urinary tract infection prophylactic agent during pregnancy. Acta Obstet. Gynecol. Scand..

